# Pathogen genomics in public health laboratories: successes, challenges, and lessons learned from California’s SARS-CoV-2 Whole-Genome Sequencing Initiative, California COVIDNet

**DOI:** 10.1099/mgen.0.001027

**Published:** 2023-06-02

**Authors:** Emily A. Smith, Kevin G. Libuit, Curtis J. Kapsak, Michelle R. Scribner, Sage M. Wright, John Bell, Christina Morales, Megan Crumpler, Sharon Messenger, Jill K. Hacker, Katya Ledin, Carol Glaser, Kathleen Jacobson, Joel R. Sevinsky, Debra A. Wadford

**Affiliations:** ^1^​ Theiagen Genomics, Highlands Ranch, Colorado, USA; ^2^​ California Department of Public Health, Richmond, California, USA; ^3^​ Orange County Public Health Laboratory, Santa Ana, California, USA

**Keywords:** whole-genome sequencing, SARS-CoV-2, bioinformatics, public health, pathogen genomics, California COVIDNet

## Abstract

The capacity for pathogen genomics in public health expanded rapidly during the coronavirus disease 2019 (COVID-19) pandemic, but many public health laboratories did not have the infrastructure in place to handle the vast amount of severe acute respiratory syndrome coronavirus 2 (SARS-CoV-2) sequence data generated. The California Department of Public Health, in partnership with Theiagen Genomics, was an early adopter of cloud-based resources for bioinformatics and genomic epidemiology, resulting in the creation of a SARS-CoV-2 genomic surveillance system that combined the efforts of more than 40 sequencing laboratories across government, academia and industry to form California COVIDNet, California’s SARS-CoV-2 Whole-Genome Sequencing Initiative. Open-source bioinformatics workflows, ongoing training sessions for the public health workforce, and automated data transfer to visualization tools all contributed to the success of California COVIDNet. While challenges remain for public health genomic surveillance worldwide, California COVIDNet serves as a framework for a scaled and successful bioinformatics infrastructure that has expanded beyond SARS-CoV-2 to other pathogens of public health importance,

## Introduction

The coronavirus disease 2019 (COVID-19) global pandemic galvanized an unprecedented expansion of microbial genomics capabilities for public health laboratories (PHLs) around the world. Throughout this expansion, PHLs were confronted with daunting challenges, including managing the massive amounts of data generated by genomic surveillance methods such as whole-genome sequencing (WGS) and setting up new bioinformatics and genomic epidemiology pipelines to allow analysis, interpretation, and formulation of actionable conclusions.

The development and subsequent distribution of amplicon-based sequencing protocols for severe acute respiratory syndrome coronavirus 2 (SARS-CoV-2) greatly assisted with method implementation. Building bioinformatics and genomic epidemiology capabilities, especially in laboratories with limited programming or bioinformatics experience, remains a challenge for many PHLs.

The genomic surveillance program implemented as part of the SARS-CoV-2 pandemic response across California, USA, offers a case study in how bioinformatics and genomic epidemiology capabilities can be scaled rapidly and applied across a large, systematically complex, resource diverse, and populous region. California has more than 25 state and local (i.e. city- and county-level) PHLs, which vary in capacity for WGS and differ in IT infrastructure and capability.

To maximize public health impact, SARS-CoV-2 genomic surveillance in California needed to be quickly implemented, the resulting sequence data needed tools for relatively easy analysis, and the data system had to be secure. The ability to ingest multiple data types and allow for hierarchical data sharing was also essential to accommodate the different public health jurisdictions in California while maintaining a centralized and standardized system. To surmount these bioinformatic challenges, the California Department of Public Health (CDPH) state PHL, in partnership with the pathogen genomics consulting company Theiagen Genomics (Highlands Ranch, CO, USA), and collaborators from local PHLs, the Chan Zuckerberg Biohub, and several academic and industry sequencing laboratories, created a comprehensive and robust laboratory network for SARS-CoV-2 genomic surveillance, known as California COVIDNet, hereafter designated as COVIDNet. The bioinformatics infrastructure of COVIDNet uses a combination of tools hosted on the Google (Mountain View, CA, USA) cloud platform that meet public health needs. Here, we describe the successful components of the COVIDNet bioinformatics infrastructure, some remaining challenges, and recommendations for PHLs to leverage this infrastructure for other pathogens of public health significanece.

## Cloud-based bioinformatics for pathogen genomics in public health

Resource scalability and the ease with which hierarchical data access and secure data sharing protocols can be applied make cloud computing an ideal resource for developing a state-wide system to support pathogen genomics. In California, cloud computing was implemented for the COVIDNet program through the adoption of Terra.bio, an open-access web application co-developed by the Broad Institute (Cambridge, MA, USA), Microsoft Corporation (Redmond, CA, USA), and Verily Life Sciences (South San Francisco, CA, USA), that connects users to scalable cloud computing resources and over 1000 open-source, containerized bioinformatics workflows via a graphical user interface. Access to Terra.bio within COVIDNet is provisioned by CDPH: users from California (CA) PHLs, including the state PHL and the 28 independent local PHLs, obtain Google login credentials through CDPH, allowing them to access the bioinformatics infrastructure. Within Terra.bio, each PHL has their own workspace, which gives individual laboratories the autonomy to conduct genomic surveillance within their jurisdiction while ensuring data can be shared with the state. Currently, the Terra.bio COVIDNet project contains over 800 000 California SARS-CoV-2 sequences, including sequences from 19 California public health laboratories as well as from the Centers for Disease Control and Prevention’s (CDC’s) contract sequencing laboratories, to maximize our understanding of the viral landscape in California.

A public workspace containing select sequences from the COVIDNet Initiative has been made available for demonstration (https://app.terra.bio/#workspaces/theiagen-demos/MGen-CDPH-2023). The demo workspace may be accessed by logging into Terra.bio using any Google account. Once in Terra, click on View Workspaces, select the ‘Public’ tab and then click on ‘Mgen-CDPH-2023’, which will give the reader access to sequence data, TheiaCov workflow outputs, available workflows, and more.

## Open-source workflows for best practices in public health bioinformatics

The use of open-source, containerized bioinformatics workflows developed by Theiagen Genomics and made available on Terra.bio allowed the rapid implementation of standardized SARS-CoV-2 analysis across all COVIDNet laboratories. Open-source workflows help ensure the transparency, reproducibility and quality of results, as the code underlying the tools is publicly available for review. The TheiaCoV workflows, hosted within the Public Health Viral Genomics (PHVG) Github repository, are used by COVIDNet laboratories to assemble and characterize SARS-CoV-2 sequence data across multiple sequencing platforms and methodologies, including Illumina (San Diego, CA, USA) single-end, Illumina paired-end, Oxford Nanopore (Oxford, UK), Clear Labs (San Carlos, CA, USA), and Element Biosciences (San Diego, CA, USA) (https://github.com/theiagen/public_health_viral_genomics). Having a set of workflows that accommodates multiple sequencing methods allowed for the standardization of outputs essential for downstream analyses and visualizations.

There are also workflows available on Terra.bio that are used by COVIDNet laboratories to prepare sequence data and metadata for submission to GISAID, the Sequence Read Archive (SRA), and GenBank, known as the Mercury workflows (https://github.com/theiagen/public_health_viral_genomics) [1, 2]. These Mercury workflows have contributed to the submission of more than 200 000 sequences from CDPH to GISAID as well as over 50 000 from local PHLs in California to GISAID. Simplifying the process of submitting data to public repositories was crucial to this effort, as the strict formatting requirements and volume of metadata needed for submission can be barriers to entry for smaller PHLs not familiar with this process. Sequence data from CDPH were also submitted to National Center for Biotechnology Information (NCBI) databases under BioProject PRJNA750736, resulting in more than 190 000 SRA submissions and 131 000 GenBank submissions. These numbers are lower than the total submissions to GISAID due to more stringent submission criteria and limitations on the types of sequencing platforms that are accepted.

## Training sessions for continued public health workforce development

While Terra.bio offers an accessible interface for laboratorians to access bioinformatics resources, training workshops and continuous support are a critical component of the COVIDNet network. Theiagen Genomics provides key training and technical support to COVIDNet Terra.bio users in the form of weekly office hours, email help line, and direct training on how to upload data, launch workflows and evaluate data quality and Pango lineage assignments [3]. Training includes genomic epidemiology basics such as how to build phylogenetic trees and use web-based tools such as Auspice [4] and UShER [5] to determine new introductions or whether sequences belong to a specific outbreak or a particular geographical origin. Weekly office hours sessions for COVIDNet Terra.bio users allow for interactive technical support, review of current and pressing topics on SARS-CoV-2 genomic characterization and analysis, workflow modifications and development of additional features in an environment of practitioner-led research and development that has been crucial to the success of COVIDNet.

## Automated alerting and visualization for real-time monitoring of genomic surveillance data

All data on Terra.bio from California are exported to Google BigQuery hourly, where they are combined and then ingested into Google Looker, a platform with alerting functionality that can notify a specified group of users when a new sequence matches specific user-defined criteria. For example, during the initial emergence of the Omicron variant, an email notification was sent to CDPH laboratory scientists and epidemiologists every time a new BA.1 sequence from one of the many different sequencing laboratories was identified. This allowed CDPH to launch epidemiological investigations in a matter of minutes following initial identification.

**Fig. 1.**
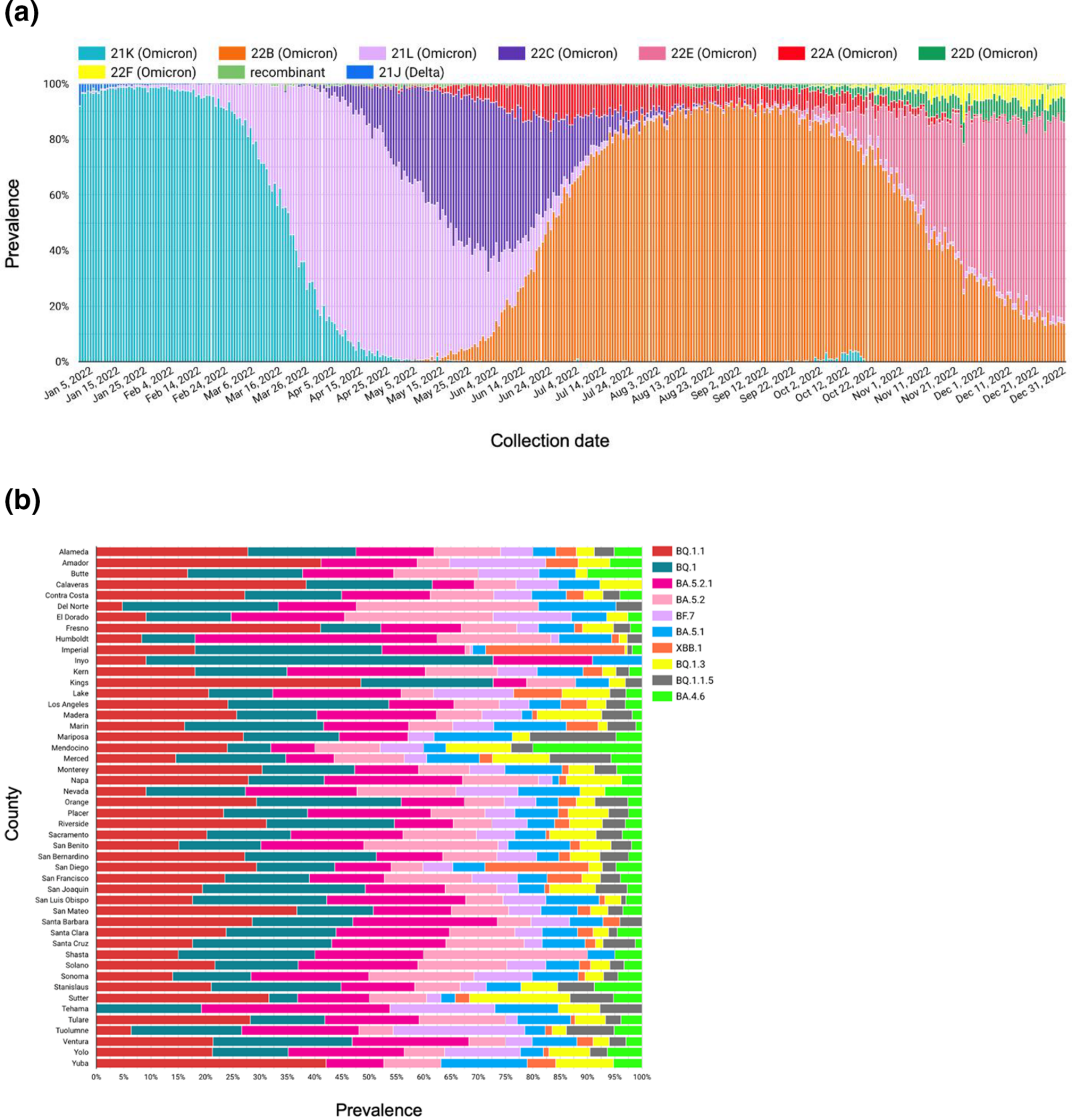
Visualizations from the SARS-CoV-2 Dashboard in Google Looker Studio. (**a**) Differences in the proportion of SARS-CoV-2 Nextclade clades by collection date in California for 2022. (**b**) Pango lineage by county from October–December 2022.

Data from Terra.bio also feed into a SARS-CoV-2 dashboard in Google Looker Studio that public health laboratory scientists, bioinformaticians and epidemiologists throughout California can access. This allows local PHLs in California to see real-time data from their jurisdiction sequenced by other laboratories such as CDPH or one of the CDC partner laboratories. Those using the dashboard can conduct searches based on different metadata or genomic characteristics, such as county, collection date, Pango lineage [3], Nextclade clade [4], quality metrics, mutations, and more (Fig. 1a, b). Public repository accession numbers are also included in the dashboard so that users can copy and paste those in the UShER web tool to view sequences on a phylogenetic tree in a matter of seconds, as opposed to the hours-long process it can traditionally take to build large-scale phylogenetic trees [5]. From there, users can determine if the sequences might be the result of an outbreak based on genetic distance, or what the geographical origins might be based on the location of closely related sequences in public repositories.

## Expansion to other pathogens of public health and future recommendations

The COVIDNet bioinformatics system has provided the cloud-based infrastructure to allow California PHLs to expand genomics to other pathogens of public health importance within the state. California PHLs can adapt the TheiaCoV workflows for use with other viral pathogens, and similarly, laboratories can import other open-source workflows for bacterial and fungal pathogen genome assembly and characterization. A critical element for the successful adoption of open-source workflows across a diverse landscape of PHLs is the ability to generate all relevant organism-specific characteristics and quality control parameters with minimal input from the user. This lowers the barrier to WGS analysis for PHLs that do not have bioinformaticians or genomic epidemiologists. However, the open-source nature of these workflows provides the flexibility to use other cloud-based platforms or a command-line approach for bioinformatics if the PHL prefers not to empoly the Terra.bio option.

As the pandemic matures and analytic tools are refined for genomic characterization, the ability of PHLs to nimbly adapt is critical for effective genomic surveillance. One component of genomic surveillance that remains a challenge for PHLs is the integration of epidemiological metadata with the sequence data. This would simplify the process of measuring trends between specific genomic characteristics and epidemiologic factors, such as age, vaccination status, and clinical outcomes. However, this integration is often complicated by the incompatibility of databases that host clinical data, epidemiological information and laboratory results, as well as necessary privacy concerns and data integrity and security. Cloud-based, HIPAA-compliant approaches will allow PHLs to overcome these obstacles in the future, particularly those with security features allowing different permission types for different users so that those who need sensitive information for epidemiological investigations can do so without the need to maintain separate systems.

The flexibility, security, and scalability of cloud-based bioinformatics and genomic epidemiology have been vital to the implementation and ongoing success of California’s COVIDNet initiative. The COVIDNet bioinformatics infrastructure provided an operational solution to sustain ongoing genomic surveillance of this novel virus, in the midst of surges from emerging variants, which generated critical data to support public health disease investigations and aid in understanding SARS-CoV-2 evolution. The COVIDNet bioinformatics infrastructure is resilient and applicable to other pathogens, including monkeypox virus and influenza virus, and serves as a model for implementing a successful bioinformatics program to support genomic surveillance within PHLs around the world.
